# Primary Central Nervous System Lymphoma Associated With Immune Deficiency and Dysregulation: Challenges in Treatment and Management

**DOI:** 10.7759/cureus.95105

**Published:** 2025-10-21

**Authors:** Yuki Dan, Akihiro Inoue, Yukihiro Miyazaki, Riko Kitazawa, Takeharu Kunieda

**Affiliations:** 1 Neurosurgery, Ehime University Graduate School of Medicine, Toon, JPN; 2 Hematology, Clinical Immunology, and Infectious Diseases, Ehime University Graduate School of Medicine, Toon, JPN; 3 Diagnostic Pathology, Ehime University Graduate School of Medicine, Toon, JPN

**Keywords:** cerebral infarction, diffuse large b-cell lymphoma, kidney transplantation, lymphomas arising in immune deficiency and dysregulation, radiation therapy

## Abstract

Lymphomas associated with immune deficiency and dysregulation (IDD) rarely occur in the central nervous system (CNS). Discontinuing immunosuppressives is the first choice of treatment, but it is often not feasible after solid organ transplantation (SOT). We report a case of primary CNS lymphoma (PCNSL) arising in IDD with an unusual clinical course. A 47-year-old woman with a history of chronic use of immunosuppressives after a kidney transplantation presented with a one-month history of headaches and left-sided weakness for two days. Gadolinium (Gd)-enhanced MRI of the brain revealed a mass lesion with ring enhancement in the right basal ganglia, and diffusion-weighted imaging (DWI) confirmed cerebral infarction in the right corona radiata. All blood tumor markers were negative, but cerebrospinal fluid analysis showed a highly elevated β2-microgrobulin (MG). PET showed relatively high 18F-fluorodeoxyglucose (FDG) uptake within the area of ring enhancement on MRI, but no obvious accumulations in tissues outside the CNS. The history and results of laboratory examinations and imaging studies suggested an initial diagnosis of malignant lymphoma. Following resection, the lesion was diagnosed histologically as diffuse large B-cell lymphoma (DLBCL). The tumor was negative for Epstein-Barr virus (EBV)-encoded small RNA; however, blood tests confirmed the presence of EBV-DNA (2.81 LogIU/mL). As the immunosuppressive therapy could not be discontinued, rituximab was initiated on the day after surgery. However, the lesion progressed rather than regressed, and postoperative radiation treatment was administered. MRI four months after surgery showed tumor shrinkage. CNS lymphoma arising in IDD should be considered when a mass lesion develops during long-term immunosuppressive therapy after transplantation.

## Introduction

Lymphomas arising in immune deficiency and dysregulation (IDD) are tumors that occur in the context of exogenous immunosuppression after solid organ transplantation (SOT) or hematopoietic stem cell transplantation. This pathology is a new entity defined in the 5th edition of the World Health Organization (WHO) classification and corresponds to immunodeficiency-associated lymphoproliferative disorders (LPDs) in the 4th edition of the WHO classification [1,2]. This entity following SOT is a rare complication, but its incidence has increased in recent years [3]. Compared with the general lymphomas of the same type that occur in healthy individuals, lymphomas arising in IDD tend to occur more frequently in organs outside the lymphatic system but rarely in the central nervous system (CNS) [3]. The treatment varies depending on the cause of IDD in the patient. Therefore, improving the patient’s immune deficiency status is an important consideration. However, discontinuing immunosuppressive agents to achieve this is extremely difficult in patients who have undergone SOT [1-3]. We report herein our experience with a case of primary CNS lymphoma arising in IDD that presented with cerebral infarction, in which diagnosis and treatment were difficult. We also present the pathological and molecular features of this entity.

## Case presentation

**Figure 1:**
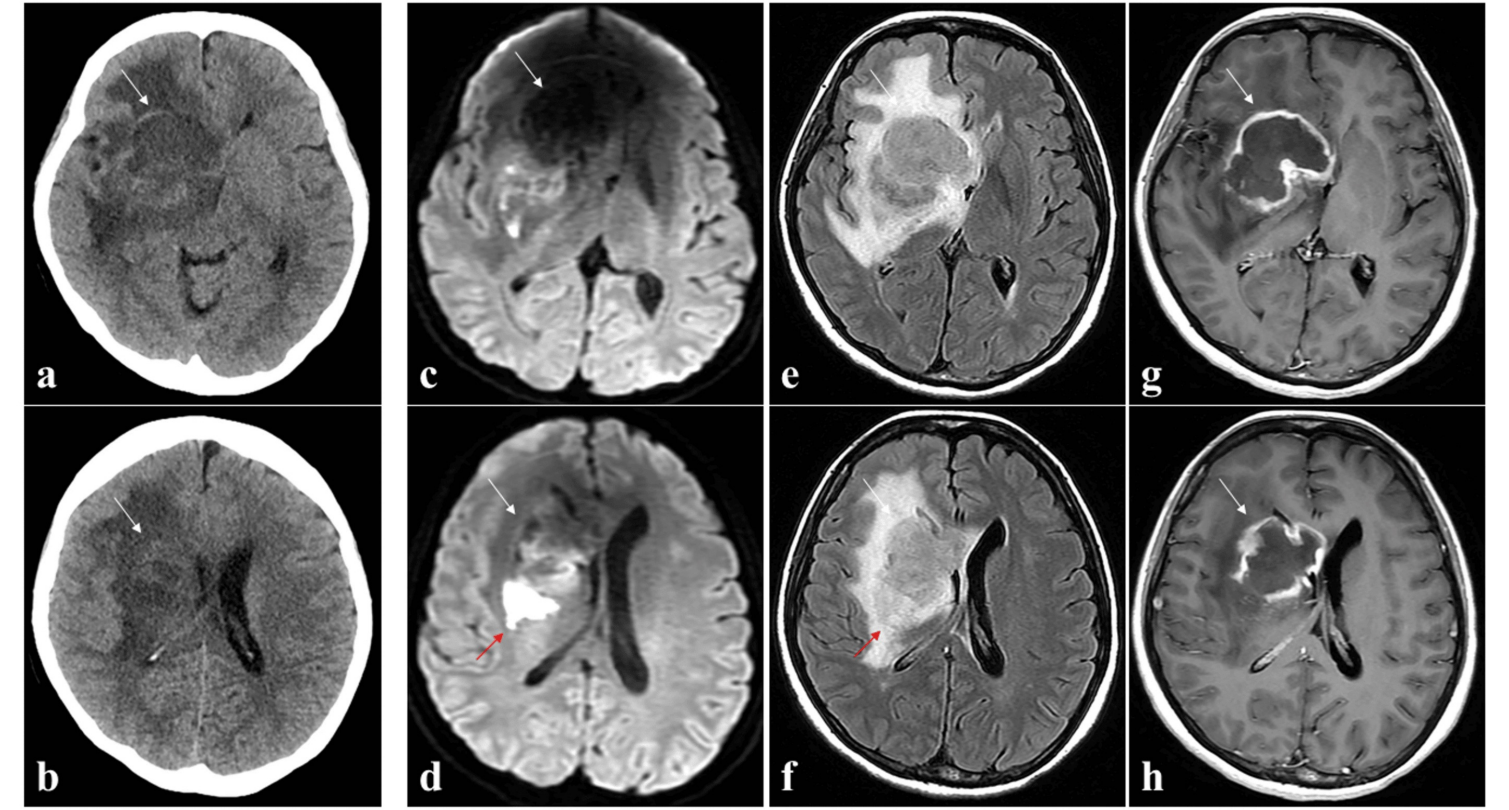
Computed tomography (CT) and magnetic resonance imaging (MRI) on admission. CT images (a, b) without contrast enhancement show an abnormal mass (white arrow) in the right frontal lobe and basal ganglia. Preoperative axial diffusion-weighted images (DWI) (c, d), fluid-attenuated inversion recovery images (FLAIR) (e, f), and gadolinium (Gd)-enhanced T1-weighted images (g, h) show an intracranial mass with ring enhancement (white arrow). In addition, the right corona radiata exhibited areas of restricted diffusion on DWI, which were interpreted as cerebral infarction (red arrow: d, f).

A 47-year-old woman with a history of using immunosuppressive drugs (tacrolimus and mycophenolate mofetil) for 11 years after a kidney transplantation presented with a one-month history of headaches and left-sided weakness that had begun two days earlier. On admission, the patient had a Karnofsky performance status (KPS) score of 60. She had severe renal impairment, with a serum blood urea nitrogen level of 48 mg/dL, a creatinine level of 4.00 mg/dL, and an estimated glomerular filtration rate (eGFR) of 10.0 mL/min/1.73 m², but did not require dialysis. Computed tomography (CT) showed an area of low density in the right hemisphere (Figures 1a, 1b) that was evident on MRI as a mass lesion hypointense on T1-weighted imaging (WI) and diffusion-weighted imaging (DWI), and hyperintense on T2-WI and fluid-attenuated inversion recovery imaging (FLAIR), with strong ring enhancement on contrast-enhanced T1-WI using gadolinium (Gd) performed unavoidably after obtaining sufficient informed consent to establish an accurate diagnosis. In addition, DWI confirmed cerebral infarction in the right corona radiata (Figure 1c-1h).

**Figure 2:**
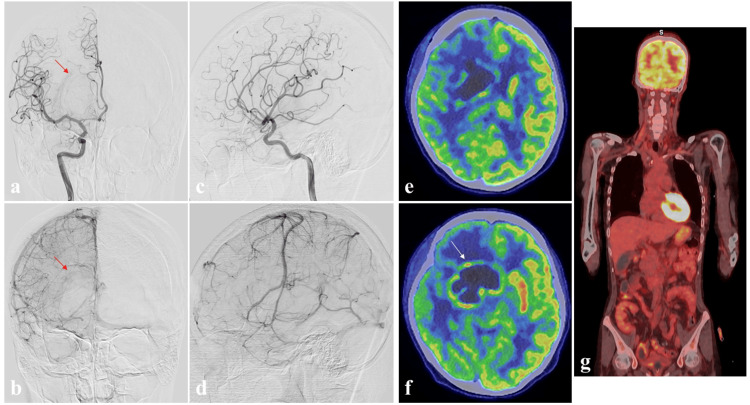
Preoperative cerebral angiography and 18F-fluorodeoxyglucose (FDG) positron emission tomography (PET). Anterior-posterior (a, b); lateral views (c, d) of right internal carotid artery angiography show slight tumor staining (red arrow), but no early venous filling. Positron emission tomography (e, f, g) shows relatively high 18F-fluorodeoxyglucose uptake within the ring-enhancing region evident on MRI (tumor-to-contralateral normal brain tissue ratio: 1.91) (white arrow), with no obvious accumulations in tissues outside the central nervous system.

Cerebral angiography showed slight tumor staining, but no early venous filling on right internal carotid angiography (Figures 2a-2d). All blood tumor markers were negative, but cerebrospinal fluid (CSF) analysis showed highly elevated β2-microgrobulin (MG) (4.0 mg/L). Relatively high 18F-fluorodeoxyglucose (FDG) uptake was seen on positron emission tomography (PET) within the ring-enhancing region on MRI (tumor-to-contralateral normal brain tissue ratio: 1.91), with no obvious accumulations in tissues outside the CNS (Figure 2e-2g).

**Figure 3:**
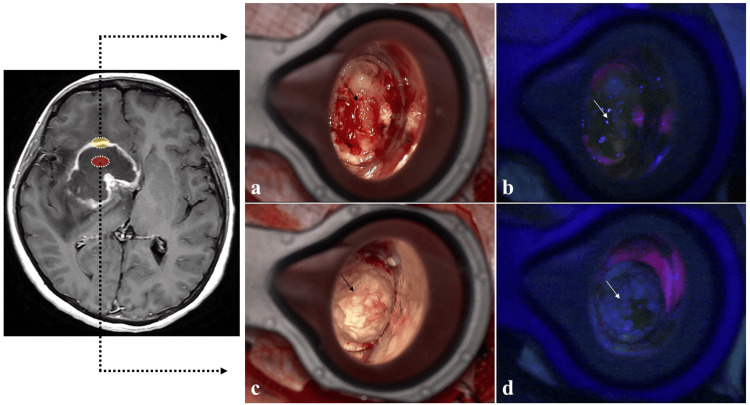
Intraoperative findings. Intraoperative findings of (a, b) gadolinium (Gd)-enhancing area (yellow oval) and (c, d) central non-enhancing area (red oval) of the lesion are of an elastic yellowish hard tumor with a clear border (black arrow), with no fluorescence on photodynamic diagnosis using 5-aminolevulinic acid (white arrow).

Initially, the history and results of the laboratory, including CSF analysis of β2-MG and imaging examinations, seemed most consistent with malignant lymphoma or glioblastoma. To obtain a histological diagnosis and plan effective treatment for the primary disease, we therefore performed a surgical biopsy of the right frontal lesion under image-guided navigation, which confirmed the presence of an elastic, yellowish, hard tumor with a clear border. The tumor showed no fluorescence by photodynamic diagnosis (PDD) using 5-aminolevulinic acid (5-ALA) (Figure 3a-3d).

**Figure 4:**
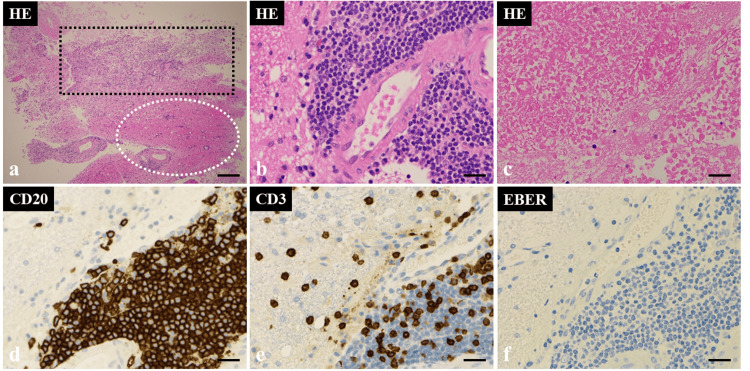
Histopathology of the biopsy specimen. Hematoxylin and eosin staining (a, b, c) reveals extensive necrosis (black dashed square area) and focal lymphocytic tumor lesions with atypical large lymphocytes with irregular nuclei (white dashed circle area). Immunohistochemical examination demonstrates cluster of differentiation (CD) 20 (+) (d) and CD 3 (–) (e), and a histological diagnosis of diffuse large B-cell lymphoma. The tumor (f) is negative for Epstein-Barr virus-encoded small RNA. a: magnification, x40; scale bar, 500 µm. b–f: magnification, x400; scale bar, 100 µm.

Histological examination with hematoxylin and eosin (HE) staining revealed extensive necrosis and focal lymphocytic tumor lesions with atypical large lymphocytes with irregular nuclei (Figures 4a-4c). Immunohistochemical examination demonstrated cluster of differentiation (CD) 20 (+) and CD 3 (-), and the histological diagnosis was diffuse large B-cell lymphoma (DLBCL) (Figures 4d, 4e). In addition, a non-germinal center B-cell (GCB)-like phenotype was identified from the results of CD10 (-), bcl-6 (-), and MUM1 (+) according to the decision tree proposed by Hans et al. [4]. The final diagnosis was CNS lymphoma arising in IDD due to her history of immunosuppressive treatment for a kidney transplant. The tumor was negative for Epstein-Barr virus (EBV)-encoded small RNA (EBER) (Figure 4f); however, blood tests confirmed the presence of EBV-DNA (2.81 LogIU/mL), indicating EBV DNA-emia.

**Figure 5:**
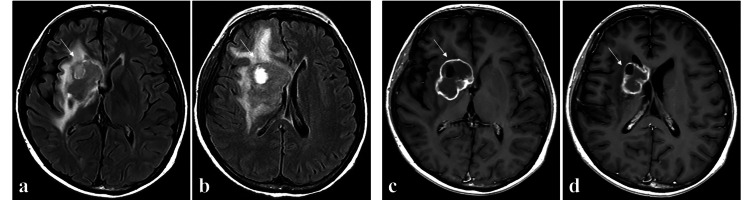
MRI acquired four months after surgery. Fluid-attenuated inversion recovery imaging (FLAIR)  (a, b) and gadolinium (Gd)-enhanced T1-weighted images (c, d) show shrinkage of the tumor (white arrow).

Despite initiation of rituximab the day after surgery (as the immunosuppressive therapy could not be discontinued), the lesions did not regress, instead showing a progressive clinical course. With consideration to her renal dysfunction, postoperative radiation therapy (whole brain: 30.0 Gy in 15 fractions) was administered. MRI acquired four months after surgery showed that the tumor had shrunk (Figure *5*).

Even now, one year after the surgery, no clear signs of recurrence have been observed. In addition, the disease state stabilized, and the patient had a KPS score of 90. The clinical study of this case was approved by the ethics committee of our institution, and informed consent was obtained from the patient.

## Discussion

Lymphomas arising in IDD exhibit diverse histopathological features [1]. As they exhibit histological and immunohistochemical findings similar to those of lymphomas occurring in healthy individuals, they are diagnosed according to the standard criteria for lymphoma. In addition, lymphomas positive for EBV or Kaposi’s sarcoma-associated herpesvirus (KSHV)/human herpesvirus-8 (HHV8) are reported to occur at a higher frequency in patients with IDD than in healthy individuals [1]. CNS lymphoma occurs in approximately 0.01% of all SOT patients, making it a rare complication [5]. Previous studies have reported incidences of 62%-79% after kidney transplantation and 10%-14% after liver transplantation [5-7], with onset more than one year after transplantation (late onset) in 95% and more than 10 years after transplantation (very late onset) in 45% [3]. CNS lymphoma has a higher incidence of late onset than non-CNS lymphoma [6], with very late onset accounting for 23%-36% of cases [6,7]. Ishihara et al. reported that the average interval between SOT and the onset of CNS lymphoma arising in IDD was 116.5 months (very long) and that 60% of cases were EBV-related [8]. The present case exhibited a very late onset pattern, with a period of 11 years between kidney transplantation and tumor onset. Although EBER by pathological study was negative, EBV DNA-emia was present, suggesting that EBV may have played a role in tumor development.

As the EBV positivity rate in biopsy specimens has been reported to exceed 90% in these tumors [9], confirming EBV DNA-emia is very important in suspected cases. However, the EBV DNA positivity rate in whole blood of CNS lymphoma arising in IDD has been reported as 27%, which is not particularly high. The low rate is probably because these lesions are confined to the CNS, resulting in low levels of EBV DNA in the blood [9]. Immunohistochemistry using EBER or EBER-in situ hybridization (ISH) has recently been used instead of serum EBV-DNA quantification for detecting EBER present in cells infected with EBV on tissue sections. EBER-ISH is useful for identifying tumors and diseases associated with latent EBV infection because it can stain EBV-infected cells more easily and with greater sensitivity compared with immunostaining. However, some cases of EBV-associated lymphoma are EBER-ISH negative. In the study of Okamoto et al., all cases with serum EBV-DNA levels of 1.0 × 10⁵ copies or higher were EBER-ISH positive, and cases with low EBV-DNA levels could be EBER-ISH negative, thus requiring caution [10]. Similarly, EBV-DNA was detected in the blood of the present case, but the serum EBV-DNA concentration was low (2.81 log IU/mL), suggesting EBV-related lymphoma despite EBER negativity, so we speculated that EBV may be involved in the development of the lymphoma.

The imaging characteristics of CNS lymphoma arising in IDD include the following: brain lesions are localized around the ventricles or basal ganglia and often present as multiple lesions; Gd contrast enhancement is commonly heterogeneous or ring-shaped; in apparent diffusion coefficient (ADC) mapping of lesions, diffusion coefficients are higher in central areas of necrosis and lower in peripheral areas with high cellular density, resulting in a heterogeneous pattern; susceptibility-weighted imaging (SWI) demonstrates features characteristic of hemorrhage, with many cases showing punctate or fine linear areas of low signal; and brain perfusion images obtained using the dynamic susceptibility contrast method show poor vascularization and no findings of hyper-perfusion [11]. Although these imaging characteristics are not necessarily specific to CNS lymphoma arising in IDD, many were observed in the present case. Therefore, in patients who exhibit atypical mass lesions after SOT, we consider that these imaging findings, along with positivity for EBV DNA-emia, are possibly diagnostic for CNS lymphoma arising in IDD.

A previous study has reported that treatment for lymphoma arising in IDD includes reducing immunosuppressive agents and administration of therapy consistent with that for primary CNS lymphoma (PCNSL), and that median overall survival (OS) is 17 months [5]. The Japanese guidelines for PCNSL recommend chemotherapy based on high-dose methotrexate (HD-MTX) therapy, with the option of combination therapy with rituximab. Whole-brain irradiation has also been recommended following chemotherapy [3]. Cavaliere et al. reported that whole-brain irradiation achieved complete or partial remission in 87.5% of cases, regardless of the use of chemotherapy, with a median OS of 27 months [6]. They reported that radiation therapy was a viable option for cases with systemic toxicity and complications due to increased susceptibility to infection and that whole-brain irradiation monotherapy may be considered in cases for which chemotherapy is not feasible [6]. Tirabrutinib, a Bruton’s tyrosine kinase (BTK) inhibitor, has recently emerged as an effective treatment option for recurrent or refractory PCNSL [12], with a study reporting the efficacy of tirabrutinib for CNS lymphoma arising in IDD. Regarding the prognosis for CNS lymphoma arising in IDD, Zimmermann et al. reported an OS rate of 63% at 2 years after onset [13], and Evens et al. reported an OS rate of 43% and a progression-free survival rate of 32% at three years after onset [7]. Mahale et al. reported an OS rate of 35% at five years after onset, with high mortality attributed to factors such as the CNS lesions themselves, as well as the approximately threefold risk of graft failure or re-transplantation due to reduction or modification of immunosuppressive agents used to control the underlying disease [5]. The present case initially received rituximab due to her renal dysfunction, but it became necessary to consider alternative treatment options after early efficacy was not observed. It was not feasible to discontinue the immunosuppressive agents, and renal dysfunction prevented implementation of therapy based on HD-MTX. Therefore, we introduced radiation therapy, which improved the neurological symptoms; however, complete remission has not yet been achieved. If disease progression is observed in the future, administration of tirabrutinib will be considered.

## Conclusions

We report a case of CNS lymphoma arising in IDD that developed 11 years after kidney transplantation. This disease should be suspected if an atypical mass lesion is observed in patients using immunosuppressive agents, and quantitative evaluation of serum EBV should be performed. If EBV DNA-emia is confirmed, lymphoma arising in IDD must be considered as one diagnostic option, and a treatment plan should be established. Further studies and accumulation of cases are needed to better understand the behavior of these tumors, to identify an optimal therapeutic plan, and to standardize the diagnostic immunohistochemical and genetic analyses.
